# Carotid Plaque Score: A Useful Tool or Futile Gadget for Risk Assessment? An Update

**DOI:** 10.31083/RCM50123

**Published:** 2026-10-23

**Authors:** David Sulman, Stefano Cacciatore, Marco Bernardi, Luigi Spadafora, Atul Pathak, Francesco Perone, Stefano Figliozzi, Ali Abdelaziz, Felice Gragnano, Paolo Calabro, Dario Mafrica, Giuseppe Andò, Michael D. Shapiro, Elad Asher, Pierre Sabouret

**Affiliations:** ^1^ACTION Study Group, Inserm UMRS1166, Heart Institute, Pitié-Salpêtrière Hospital, Sorbonne University, 75013 Paris, France; ^2^Department of Physiology and Aging, College of Medicine, University of Florida, Gainesville, FL 32611, USA; ^3^UOC UTIC Emodinamica e Cardiologia, Santa Maria Goretti Hospital, 04100 Latina, Italy; ^4^Department of Medical-Surgical Sciences and Biotechnologies, Sapienza University of Rome, 04100 Latina, Italy; ^5^National Institute of Cardiology, Cardiovascular Surgery and Interventional Cardiology (INCCI), 1210 Luxembourg City, Luxembourg; ^6^Cardiac Rehabilitation Unit, Rehabilitation Clinic ‘Villa delle Magnolie’, 81020 Castel Morrone, Caserta, Italy; ^7^IRCCS Humanitas Research Hospital, 20089 Rozzano, Milan, Italy; ^8^Department of Biomedical Sciences, Humanitas University, 20072 Milan, Italy; ^9^Faculty of Medicine, Alexandria University, 5372066 Alexandria, Egypt; ^10^Department of Translational Medical Sciences, University of Campania “Luigi Vanvitelli”, 81100 Caserta, Italy; ^11^Department of Clinical and Experimental Medicine, University of Messina and Azienda Ospedaliera Papardo, 98124 Messina, Italy; ^12^Center for Prevention of Cardiovascular Disease, Department of Cardiovascular Medicine, Wake Forest University School of Medicine, Winston-Salem, NC 27157, USA; ^13^Jesselson Integrated Heart Center, Shaare Zedek Medical Center, Eisenberg R&D authority and Faculty of Medicine, Hebrew University of Jerusalem, 9103102 Jerusalem, Israel

**Keywords:** carotid plaque score, carotid ultrasonography, carotid atherosclerosis, plaque burden, subclinical atherosclerosis, cardiovascular risk assessment, risk reclassification, stroke prevention

## Abstract

Conventional cardiovascular risk scores estimate the probability of future events; however, these risk scores do not directly quantify the presence or extent of subclinical atherosclerosis. This narrative review critically evaluates the current evidence on the carotid plaque score (CPS), a semiquantitative ultrasound-based measure of carotid atherosclerotic burden across predefined arterial segments. Population-based cohorts have consistently shown that carotid plaque presence and burden are independently associated with cardiovascular and cerebrovascular events. Studies specifically evaluating CPS have further suggested an incremental prognostic value beyond conventional risk models, particularly for ischemic stroke and major adverse cardiovascular event (MACE). However, CPS does not represent a single standardized metric. Therefore, proposed thresholds, including CPS ≥2 and CPS >3, were derived using different plaque definitions, arterial segments, scoring methods, and study populations and are not directly interchangeable. Meanwhile, CPS is more accessible, non-ionizing, and relatively inexpensive compared with coronary artery calcium (CAC), but it is less extensively validated and less standardized. Nonetheless, available comparative evidence indicates that CAC provides superior discrimination for coronary outcomes. In contrast, carotid plaque assessment may offer complementary information for cerebrovascular risk and in selected individuals with discordant estimated risk or no detectable coronary calcification. No randomized trial has demonstrated that treatment decisions guided by CPS improve clinical outcomes. Accordingly, current guidelines generally position carotid plaque imaging as a selective risk modifier rather than a routine screening or stand-alone treatment-allocation tool. Thus, broader clinical implementation will require standardized acquisition and reporting protocols, external validation across diverse populations, integration with established risk models, and prospective studies evaluating whether CPS-guided preventive strategies improve patient outcomes.

## 1. Introduction

Cardiovascular risk prediction is central to primary prevention because decisions regarding lipid-lowering, antihypertensive, and other preventive interventions depend largely on the estimated probability of future cardiovascular events [1]. Contemporary algorithms, including the Systematic Coronary Risk Evaluation 2 (SCORE2) model and the American Heart Association Predicting Risk of Cardiovascular Disease EVENTs (PREVENT) equations, have improved cardiovascular risk estimation [2,3]. SCORE2 was derived from 45 cohorts across 13 countries and externally validated in 25 cohorts from 15 European countries [2], whereas PREVENT was developed using data from approximately 3.3 million U.S. adults and incorporates cardiovascular, kidney, and metabolic variables [3]. A recent multinational validation involving 6.4 million individuals confirmed generally good discrimination and calibration for both models across geographic regions [4]. Nevertheless, these tools remain probabilistic and do not directly quantify the presence or extent of subclinical atherosclerosis. This may result in discordance between estimated risk and underlying disease burden. For example, SCORE2 has been shown to overestimate cardiovascular risk in individuals without carotid plaque and to underestimate it in those with established plaque [5].

Non-invasive imaging may complement conventional risk assessment by directly capturing the cumulative vascular effects of lifetime exposure to cardiovascular risk factors. Coronary artery calcium (CAC) scoring is currently the most extensively validated imaging marker for risk reclassification and is increasingly incorporated into preventive decision-making [1]. In the Multi-Ethnic Study of Atherosclerosis (MESA), a CAC score of 0 was the strongest negative risk marker among 13 evaluated markers and provided the greatest downward risk reclassification for coronary heart disease [6]. However, CAC assessment requires computed tomography, involves ionizing radiation, and primarily detects calcified coronary plaque [7,8,9]. Carotid ultrasonography represents a widely available, non-ionizing, and comparatively inexpensive alternative that allows assessment of plaque presence, distribution, and burden [10]. Among MESA participants with CAC = 0, carotid plaque was present in 36% and was independently associated with incident coronary heart disease and subsequent CAC development [11]. Carotid plaque assessment should also be distinguished from carotid intima-media thickness (CIMT), which has shown more limited incremental value for individual risk prediction [10].

Within carotid imaging, the carotid plaque score (CPS) has emerged as a pragmatic semiquantitative approach that summarizes plaque involvement across predefined arterial segments. The American Society of Echocardiography (ASE) recommends that, when a plaque score is calculated, assessment should include the distal common carotid artery, carotid bulb, and proximal internal carotid artery [10]. Population-based studies have associated greater carotid plaque burden with ischemic stroke and cardiovascular events beyond conventional risk factors and established risk algorithms [12,13,14]. In the Akershus Cardiac Examination 1950 cohort (ACE 1950), each unit increase in CPS was associated with a higher risk of ischemic stroke and major adverse cardiovascular event (MACE) after adjustment for SCORE2 [12]. Other cohorts, including the Three-City and ARteris Cardiovascular Outcome (ARCO) studies, have shown improved risk stratification when measures of plaque distribution or total plaque area were added to conventional risk assessment [13,14].

However, CPS does not refer to a single standardized metric. ACE 1950 assigned a score of 0–3 to the largest plaque in each of eight carotid segments according to its maximal diameter and summed the segment scores to obtain a total CPS ranging from 0 to 24; a CPS >3 was identified as a high-risk threshold [12], whereas the Rotterdam Study used a site-based 0–6 scale [15,16], and the Three-City Study classified participants according to the number of plaque-positive sites [13]. These approaches differ in plaque definition, anatomical coverage, scoring procedure, study population, and outcome selection. Their numerical thresholds are therefore not directly interchangeable, limiting cross-study comparisons and the identification of a single clinically applicable cutoff.

The clinical position of CPS also remains uncertain relative to CAC. In MESA, CAC was a stronger predictor of coronary heart disease and composite cardiovascular outcomes than CPS, whereas the two modalities showed more comparable performance for stroke and transient ischemic attack prediction [17,18]. Additional comparisons in MESA, REFINE-Reykjavik, and BioImage similarly indicate that CAC provides superior discrimination for coronary outcomes, while carotid plaque assessment may offer complementary information on cerebrovascular and systemic atherosclerotic burden [11,17,19,20,21]. However, direct comparisons remain limited, particularly in Asian, African, and Latin American populations, and no randomized trial has compared CPS-guided with CAC-guided preventive strategies. These uncertainties are reflected in the heterogeneous positions adopted by cardiovascular and imaging societies [1].

Therefore, this narrative review aims to critically synthesize the current evidence on CPS, distinguish it from related carotid plaque burden measures, compare the principal scoring approaches and proposed thresholds, examine methodological and geographic sources of heterogeneity, evaluate its incremental value relative to conventional risk models and established imaging modalities, and identify priorities for standardization, implementation, and future interventional research.

## 2. Methods and Literature Search

This narrative review provides an updated and critical overview of the role of CPS and carotid plaque imaging in cardiovascular risk assessment. A targeted literature search was conducted in PubMed/MEDLINE from database inception through July 2026 using combinations of the following terms and Boolean operators: “carotid plaque score”, “carotid plaque burden”, “carotid ultrasonography”, “carotid atherosclerosis”, “cardiovascular risk prediction”, “risk reclassification”, “stroke prevention”, “coronary artery calcium”, and “plaque vulnerability”. English-language guidelines, consensus documents, scientific statements, cohort and comparative studies, systematic reviews, and meta-analyses were considered. The reference lists of relevant publications were also reviewed to identify additional studies. Studies were selected according to their relevance to the main domains of the review, including CPS definitions and scoring methods, prognostic value, comparison with CIMT, CAC, and established risk models, guideline recommendations, clinical implementation, and future research priorities. Priority was given to longitudinal studies reporting clinically relevant cardiovascular or cerebrovascular outcomes, studies directly comparing carotid plaque assessment with established risk models or imaging modalities, and recent guidance documents from major cardiovascular and imaging societies. Cross-sectional and imaging studies were included when they provided relevant information on scoring methodology, plaque characterization, geographic representation, or implementation. Evidence was synthesized narratively. Given the narrative design, no formal risk-of-bias tool or quantitative evidence synthesis was applied. Nevertheless, the included studies were critically appraised according to study design, population characteristics, sample size, plaque definition, imaging methodology, follow-up duration, reported outcomes, and key limitations.

## 3. Carotid Imaging and Cardiovascular Risk

The prognostic relevance of carotid plaque in population-based settings was demonstrated early by the Rotterdam Study, which showed that ultrasound-detected carotid plaque was independently associated with incident stroke and coronary events beyond traditional cardiovascular risk factors [15]. In the same cohort, a subsequent high-resolution magnetic resonance imaging (MRI) substudy involving 1349 older adults demonstrated that intraplaque hemorrhage was independently associated with incident stroke and coronary heart disease, whereas plaque size and luminal stenosis provided more limited prognostic information [22]. Consistent with this broader perspective, Brinjikji et al. [23] emphasized that luminal stenosis alone may not adequately capture cerebrovascular risk and that assessment of plaque burden and composition may provide additional prognostic information. These findings contributed to a shift from the assessment of stenosis severity toward a broader evaluation of plaque burden and composition.

Subsequent ultrasound-based cohorts showed that quantitative measures of carotid plaque burden provide prognostic information beyond conventional risk factors. In the Northern Manhattan Study, maximum carotid plaque thickness of at least 1.9 mm was associated with a higher risk of ischemic stroke, myocardial infarction, or vascular death during a mean follow-up of 6.9 years [24]. Similarly, the Tromsø Study showed that total plaque area was a stronger predictor of myocardial infarction and first-ever ischemic stroke than CIMT, with particularly strong associations with myocardial infarction among women [25,26]. In the Atherosclerosis Risk in Communities (ARIC) study, adding plaque presence to conventional risk factors and CIMT improved coronary risk classification, particularly among individuals at intermediate estimated risk [27].

Other studies evaluated related measures of plaque burden rather than a formal CPS. In the Three-City Study, plaque involvement at two or more carotid sites was associated with an approximately twofold higher risk of incident coronary heart disease and improved risk reclassification [13]. In a cross-sectional analysis of the Carotid Plaque Length Registry, maximal plaque length was independently associated with coronary artery disease severity and provided greater discriminatory value than plaque score or CIMT for identifying severe coronary disease [28]. Similarly, in the cross-sectional Intimal Carotid Evaluation Before Echocardiography Reveals Global Vascular Risk (ICEBERG) study, carotid plaque was detected in more than 60% of statin-naïve individuals without known cardiovascular disease who had been classified as being at low or intermediate risk by conventional models [29]. These findings illustrate potential discordance between estimated cardiovascular risk and directly measured atherosclerotic burden, but do not establish prospective associations with clinical events.

Among studies evaluating formal CPS approaches, the ACE 1950 cohort showed that each unit increase in its diameter-based score was associated with a higher risk of ischemic stroke and MACE after adjustment for SCORE2, while a score greater than 3 identified a subgroup at particularly elevated risk [12]. However, this threshold was derived from the sum of maximal plaque diameters across carotid segments and cannot be directly extrapolated to scoring systems based on the number of plaque-positive sites.

Direct comparisons with other imaging markers provide a more nuanced interpretation of carotid plaque assessment. In MESA, CAC was a stronger predictor of coronary heart disease and composite cardiovascular outcomes than CPS, whereas the two modalities showed more similar predictive performance for stroke and transient ischemic attack [17]. Importantly, carotid plaque remained associated with incident coronary events and subsequent development of coronary calcification among individuals with a coronary artery calcium score of zero [11]. Together, these findings suggest that carotid imaging may provide complementary rather than equivalent information, particularly for cerebrovascular risk assessment and in individuals without detectable coronary calcification.

Overall, these studies support the prognostic relevance of carotid plaque presence, burden, and composition. Nevertheless, most available evidence derives from observational cohorts using heterogeneous plaque definitions, anatomical segments, imaging protocols, and outcome measures. Moreover, plaque presence, total plaque area, plaque thickness, plaque length, formal scoring systems, and vulnerability features represent related but distinct constructs and should not be considered interchangeable. This heterogeneity limits direct comparison across studies and remains a major obstacle to the standardized clinical interpretation and application of carotid plaque imaging.

## 4. From Detection to Quantification: The Carotid Plaque Score

The studies reviewed in the preceding section consistently support the prognostic relevance of carotid plaque burden. However, they also illustrate a fundamental methodological challenge: the term “carotid plaque score” does not refer to a single standardized metric. CPS can be broadly defined as an ultrasound-derived composite index intended to summarize the extent or distribution of atherosclerotic plaque across predefined carotid arterial segments. In the segment-based approach applied in the Rotterdam Study [15], one point is assigned for each of six predefined sites, comprising the common carotid artery, carotid bulb, and internal carotid artery bilaterally, yielding a score ranging from 0 to 6 (Fig. 1, Ref. [15]).

**Fig. 1.**
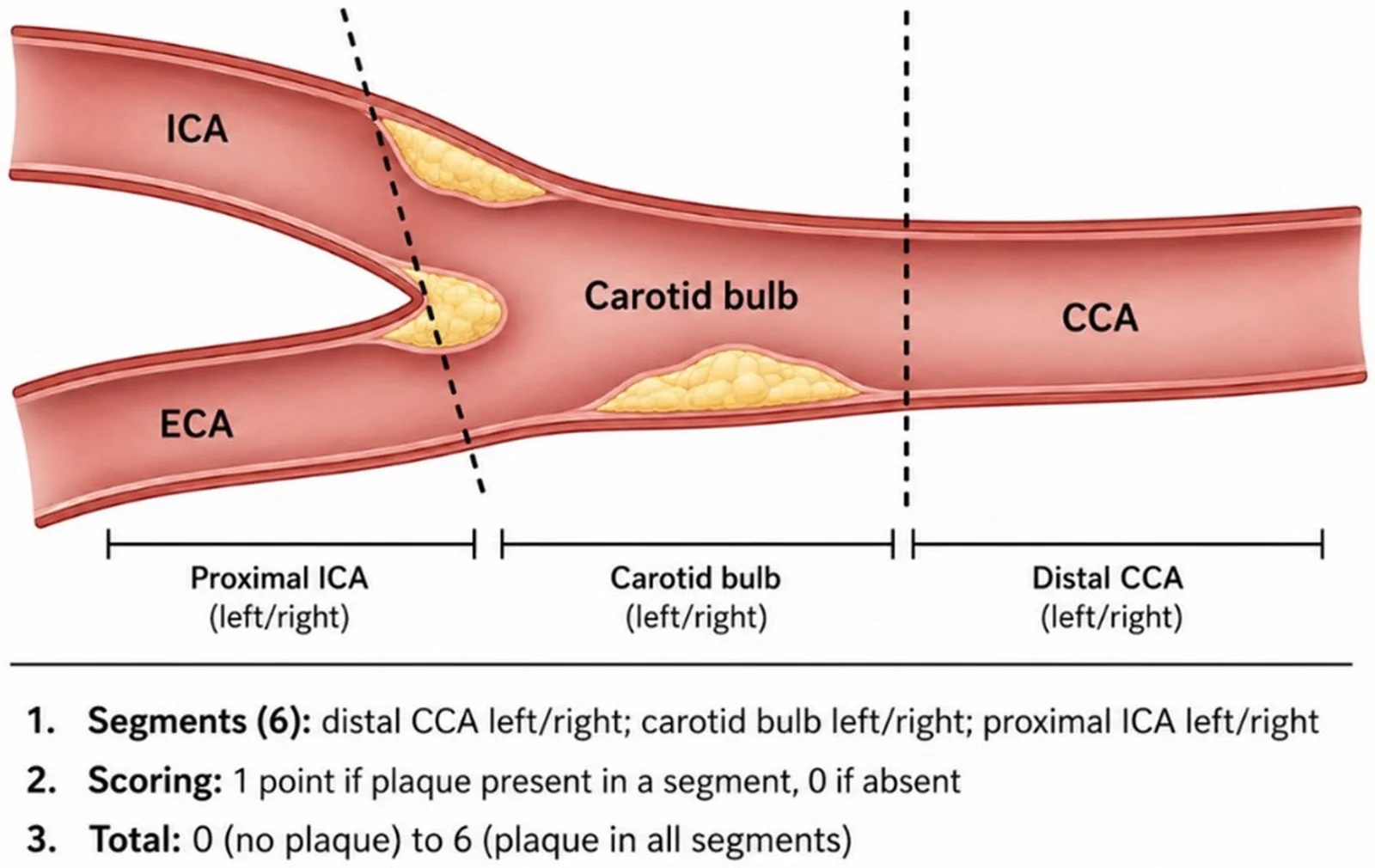
**Calculation of the Rotterdam carotid plaque score**. Created by the authors based on the Rotterdam scoring approach [15]. Abbreviations: CCA, common carotid artery; ECA, external carotid artery; ICA, internal carotid artery. Figure created by BioRender.

### 4.1 Scoring Approaches and Methodological Variability

Carotid plaque burden can be quantified using different approaches that capture related but distinct dimensions of carotid atherosclerosis and generate values and thresholds that are not directly comparable. In this review, CPS refers to composite indices that integrate plaque findings across predefined arterial segments, whereas dimensional measurements are considered related plaque burden indices. Table 1 (Ref. [12,13,15,24,25,26,28]) summarizes the main scoring approaches and related plaque burden indices reported in the literature. The principal sources of methodological variability include the plaque characteristic being quantified, the number and anatomical definition of the segments assessed, the ultrasound definition of plaque, the use of unilateral or bilateral scanning, and whether the final measure is expressed as a categorical or continuous variable [12,13,15,24,25,26,27,28].

**Table 1.** **Comparison of carotid plaque scores and related plaque burden indices**.

Approach	Construct quantified	Measurement method	Segments assessed	Output	Formal CPS	Representative studies
Segment-count score	Distribution of plaque across predefined sites	One point for each plaque-positive segment	CCA, bulb, and ICA bilaterally	0–6	Yes	Rotterdam Study [15]
Diameter-sum score	Combined plaque dimensions across segments	Sum of segment scores based on maximal plaque diameter	CCA, carotid bifurcation/bulb, ICA, and ECA bilaterally	0–24	Yes	ACE 1950 [12]
Number of plaque-positive sites	Extent of anatomical involvement	Count of sites containing plaque	Study-specific carotid sites	0, 1, or ≥2	Related score	Three-City Study [13]
Total plaque area	Overall two-dimensional plaque burden	Sum of measured plaque areas	Unilateral or bilateral carotid arteries	Continuous, mm^2^	No	Tromsø Study [25,26]
Maximal plaque length	Longitudinal extent of the largest plaque	Longest plaque measurement	Accessible carotid segments	Continuous, mm	No	CPL Registry [28]
Maximal plaque thickness	Maximum focal plaque burden	Largest plaque thickness	Accessible carotid segments	Continuous, mm	No	Northern Manhattan Study [24]

Abbreviations: ACE, Akershus Cardiac Examination; ARIC, Atherosclerosis Risk in Communities; CCA, common carotid artery; CPL, Carotid Plaque Length; CPS, carotid plaque score; ICA, internal carotid artery.

The Rotterdam Study used a segment-count approach ranging from 0 to 6 and categorized participants according to the number of plaque-positive sites [15]. By contrast, the ACE 1950 cohort used a continuous score based on the sum of maximal plaque diameters across carotid segments and identified CPS >3 as a threshold associated with increased cardiovascular risk [12]. Consequently, a value greater than 3 in ACE 1950 is not equivalent to a score of 3 on the Rotterdam scale.

Other studies assessed related but methodologically distinct measures of plaque burden. The Three-City Study classified participants according to the number of plaque-positive sites without applying the same formal scoring method used in Rotterdam or ACE 1950 [13]. The Carotid Plaque Length (CPL) Registry evaluated maximal plaque length [28], the Tromsø Study measured total plaque area [25,26], and the Northern Manhattan Study used maximal plaque thickness [24]. These approaches capture different aspects of plaque burden, and their values and proposed prognostic thresholds should not be compared directly.

The American Society of Echocardiography recommends that, when a plaque score is calculated, assessment should include the distal 1 cm of the common carotid artery, the carotid bulb, and the proximal 1 cm of the internal carotid artery [10]. The Mannheim consensus defines plaque as a focal structure encroaching into the arterial lumen by at least 0.5 mm, by at least 50% of the surrounding intima-media thickness, or as a focal thickness greater than 1.5 mm [30]. However, neither this plaque definition nor a single acquisition and scoring protocol has been applied consistently across CPS studies, further limiting comparability.

### 4.2 Sources of Heterogeneity Across Studies

In addition to differences in scoring methodology, several characteristics of the available evidence contribute to heterogeneity and limit the generalizability of proposed CPS thresholds.

#### 4.2.1 Population Characteristics and Geographic Representation

Most longitudinal CPS studies were conducted in European or North American populations, frequently including middle-aged or older adults. Plaque prevalence is strongly dependent on age, cardiovascular risk profile, ethnicity, and geographic setting [31]. Studies such as the China Kadoorie Biobank [32,33], Cardiovascular Risk Factor Multiple Evaluation in Latin America (CARMELA) study [34,35], Brazilian Longitudinal Study of Adult Health (ELSA-Brasil) [36,37], and the Africa Wits-INDEPTH Partnership for Genomic Studies (AWI-Gen), conducted within the Human Heredity and Health in Africa (H3Africa) initiative [38,39] have demonstrated substantial variation in carotid atherosclerosis across populations. However, several of these studies assessed plaque presence or carotid intima-media thickness rather than a validated CPS. They therefore document geographic variation in carotid atherosclerosis and gaps in the available evidence but do not provide direct validation of population-specific CPS thresholds.

#### 4.2.2 Ethnic and Regional Differences

Evidence regarding ethnic differences in CPS performance remains limited. The Chinese Atherosclerosis Risk Evaluation II (CARE II) study identified a high prevalence of MRI-defined high-risk plaque characteristics among Chinese patients with recent stroke or transient ischemic attack, including many high-risk plaques in arteries with less than 50% stenosis [40]. However, CARE II enrolled symptomatic patients and used magnetic resonance imaging rather than an ultrasound-based CPS [40]. Similarly, the Chinese CPL Registry found that maximal plaque length was strongly associated with coronary disease severity and provided greater discrimination than plaque score or intima-media thickness [28]. These studies suggest that plaque prevalence, morphology, and the performance of individual plaque measurements may vary across populations. However, differences in study design, clinical setting, imaging modality, and outcome assessment prevent the derivation of ethnicity-specific or region-specific CPS thresholds. Although MESA included a multi-ethnic cohort [19,41], ethnicity-stratified analyses of CPS performance remain limited.

#### 4.2.3 Ultrasound Protocols and Operator Dependence

Carotid ultrasonography is influenced by image acquisition, equipment settings, anatomical coverage, plaque definition, and reader experience. The ELSA-Brasil reproducibility substudy reported excellent intra-observer and inter-observer agreement for plaque scoring within a standardized research protocol using trained and certified operators [37]. Nevertheless, reproducibility in routine clinical practice may be lower [10,26]. Although professional societies recommend standardized scanning protocols [10,30], a widely implemented certification and quality-assurance framework specifically for CPS assessment is currently lacking.

#### 4.2.4 Study Design and Outcome Assessment

The available evidence includes prospective population-based cohorts with adjudicated cardiovascular outcomes, cross-sectional studies using surrogate or angiographic endpoints, and studies involving both asymptomatic and symptomatic populations. Longitudinal cohorts such as Rotterdam [15], ACE 1950 [12], Three-City [13], Tromsø [25,26], Northern Manhattan Study [24], ARIC [27], and MESA [11,41] provide the strongest evidence for prognostic associations. By contrast, ICEBERG [29], and the CPL Registry [28] provide information on plaque detection and concurrent coronary disease severity but cannot establish temporal associations with future cardiovascular events. CARE II [40] provides relevant information on plaque characteristics in symptomatic patients but did not evaluate an ultrasound-based CPS.

### 4.3 Summary of Cohort Evidence

Table 2 (Ref. [12,13,15,17,22,24,25,27,28,29,34,36,38,40,42,43]) summarizes the main studies evaluating formal carotid plaque scores or related plaque burden indices. Among studies specifically evaluating CPS, ACE 1950 showed that each unit increase in its diameter-based score was associated with a 25% higher risk of ischemic stroke and a 21% higher risk of MACE after adjustment for SCORE2 [12]. Segment-based and site-count approaches also provided prognostic or risk-reclassification information in the Rotterdam [15,16], Three-City [13], ARIC [27], and MESA [17,41] cohorts, although the magnitude of association and proposed thresholds varied considerably.

**Table 2.** **Major studies evaluating carotid plaque scores and related carotid plaque burden indices**.

Study	Design	Population ± follow-up	Plaque assessment	Main outcomes	Key effect estimates	Comments
Rotterdam Study [15]	Population-based prospective cohort	n = 4217; age ≥55 years; Netherlands; mean follow-up 5.2 years	US: plaque presence at six carotid sites; segment-count CPS ranging from 0 to 6	Incident stroke and cerebral infarction	Increase in total plaque score: RR 1.48 (95% CI 1.14–1.93) for stroke and RR 1.52 (95% CI 1.04–2.20) for cerebral infarction	Older, asymptomatic Dutch population; focused primarily on stroke outcomes; CPS thresholds were not externally validated
Rotterdam MRI Substudy [22]	Population-based prospective MRI substudy	n = 1349; mean age 72 years; Netherlands; median follow-up 5.1 years for stroke and 4.8 years for CHD	MRI: IPH, LRNC, calcification, maximal plaque thickness, and stenosis	Incident stroke and CHD	IPH: HR 2.42 (95% CI 1.30–4.50) for stroke and HR 1.95 (95% CI 1.20–3.14) for CHD	Included participants with US-detected subclinical carotid atherosclerosis and no previous stroke or CHD; evaluated plaque composition rather than an ultrasound-based CPS
ACE 1950 [12]	Prospective cohort	n = 3650; mean age 64; Norway; excluded prior stroke/MI ~7 yrs	US: maximal plaque diameter scored 0–3 in each of 8 carotid segments; segment scores summed to obtain CPS (0–24)	Ischemic stroke, MACE	HR 1.25 (stroke; 95% CI 1.15–1.36), HR 1.21 (MACE; 95% CI 1.14–1.27); CPS >3 high-risk	Diameter-sum method not comparable to binary CPS; single Norwegian cohort; threshold specific to this scoring system
Three-City Study [13]	Prospective cohort	n = 5895; age 65–85; France; CHD-free Median 5.4 yrs	US: number of plaque-positive sites (0, 1, ≥2)	Incident CHD	HR 2.2 (≥2 sites; 95% CI 1.6–3.1); NRI 13.7%	Older French population; did not use formal CPS; limited to CHD outcomes
Tromsø Study [25]	Population-based cohort	n = 6226; age 25–84; Norway 6–10 yrs	US: total plaque area (right carotid only)	MI, ischemic stroke	MI: RR 1.56 (men) and RR 3.95 (women), highest plaque area tertile vs no plaque.	Unilateral assessment only; TPA not directly comparable to segment-based CPS
Northern Manhattan Study [24]	Prospective cohort	n = 2189; multi-ethnic; mean age ~69; New York Mean 6.9 yrs	US: max plaque thickness	Combined vascular events	HR 2.80 (thickness ≥1.9 mm; 95% CI 2.04–3.84)	Single-measure approach; urban multi-ethnic cohort
ARIC [27]	Prospective cohort	n = 13,145; age 45–64; US; multi-ethnic Median ~15 yrs	US: plaque presence (binary) + CIMT	CHD	NRI ~9.9% for plaque + CIMT vs CIMT alone	Binary plaque assessment only; no formal CPS
MESA [17]	Prospective cohort	n = 4955; age 45–84; US; multi-ethnic Mean 11.3 yrs	US: CPS (0–12; 1 point per plaque-positive near/far wall of the CCA, bulb, and ICA bilaterally) + CT: CAC	CVD, CHD, stroke/TIA	CAC: HR 2.09 (CHD); CPS: HR 1.35 (CHD); similar for stroke	Direct CAC vs CPS comparison; CAC superior for coronary events; multi-ethnic but no ethnicity-stratified CPS analysis
ICEBERG [29]	Cross-sectional	n = 560; statin-naïve; Canada; excluded known CVD	US: carotid plaque presence according to ARIC criteria	Risk reclassification	>60% had plaque despite low–intermediate FRS	No prospective outcome data; cross-sectional assessment of carotid plaque prevalence and risk reclassification
CPL Registry [28]	Cross-sectional registry-based cohort	n = 2149; China; referred for coronary angiography	US: plaque length + CPS	CAD severity (Gensini score)	Plaque length AUC 0.819 vs CPS 0.769 vs IMT 0.634	Referral bias; cross-sectional; Chinese population—plaque length may be more informative than CPS in this setting
CARE II [40]	Cross-sectional, multicenter study	n = 1047; mean age approximately 62 years; China; patients with recent stroke or TIA	MRI: IPH, surface disruption, and LRNC occupying >40% of the vessel wall area; high-risk plaque defined by the presence of ≥1 of these features	Prevalence and distribution of high-risk carotid plaque features	High-risk plaque prevalence 28%, compared with 19% for severe stenosis ≥50%	Symptomatic population; cross-sectional study with no prospective outcome assessment; MRI-based and not an ultrasound CPS study; findings cannot be extrapolated directly to primary prevention
Nicolaides et al. [42]	Prospective cohort	n = 985; mean age 58; Cyprus; asymptomatic; stenosis <50% 13 yrs	US: plaque area, thickness, number of bifurcations with plaque	ASCVD events	NRI up to 16.6%	Long follow-up but moderate sample size; single-center; included femoral arteries
PARISK [43]	Prospective cohort	n = 244; mean age 68; Netherlands; symptomatic stenosis <70% 5.1 yrs	MRI + CTA: IPH, ulceration, plaque volume	Recurrent ipsilateral stroke/TIA	HR 2.12 (IPH); HR 1.07 per 100 µL (volume)	Small sample; symptomatic patients only; not generalizable to primary prevention
ELSA-Brasil [36]	Prospective cohort, cross-sectional baseline analysis	n = 4266 (baseline); age 35–74; Brazil; multi-ethnic	US: plaque presence + plaque score (number of sites) + CIMT	Plaque prevalence, risk factor associations	Plaque prevalence 35.9%; hypertension OR 1.39, hypercholesterolemia OR 1.58, smoking OR 2.00	Cross-sectional plaque data at baseline; outcome data pending; largest Latin American cohort with CPS
CARMELA [34]	Cross-sectional, population-based	n = 11,550; age 25–64; 7 Latin American cities	US: CIMT + plaque presence	Plaque prevalence, risk factor associations	Overall plaque prevalence 8% (range 5–14% across cities)	No outcome data; younger population; plaque prevalence much lower than European cohorts; no formal CPS
H3Africa AWI-Gen [38]	Cross-sectional, population-based	n ≈ 9000; age 40–60; Burkina Faso, Ghana, Kenya, South Africa	US: CIMT only (no plaque score)	CIMT determinants	Mean CIMT 640 ± 123 µm; carotid atherosclerosis	CIMT only, no plaque assessment; no cardiovascular outcome data; highlights gap in African CPS evidence

Abbreviations: ACE, Akershus Cardiac Examination; ARIC, Atherosclerosis Risk in Communities; ASCVD, atherosclerotic cardiovascular disease; AUC, area under the curve; AWI-Gen, Africa Wits-INDEPTH Partnership for Genomic Studies; CAC, coronary artery calcium; CAD, coronary artery disease; CARE II, Chinese Atherosclerosis Risk Evaluation II; CARMELA, Cardiovascular Risk Factor Multiple Evaluation in Latin America; CHD, coronary heart disease; CIMT, carotid intima-media thickness; CPL, Carotid Plaque Length; CPS, carotid plaque score; CT, computed tomography; CTA, computed tomography angiography; CVD, cardiovascular disease; ELSA-Brasil, Brazilian Longitudinal Study of Adult Health; FRS, Framingham Risk Score; H3Africa, Human Heredity and Health in Africa; HR, hazard ratio; ICEBERG, Intimal Carotid Evaluation Before Echocardiography Reveals Global Vascular Risk; IPH, intraplaque hemorrhage; LRNC, lipid-rich necrotic core; MACE, major adverse cardiovascular event; MESA, Multi-Ethnic Study of Atherosclerosis; MI, myocardial infarction; MRI, magnetic resonance imaging; NRI, net reclassification improvement; OR, odds ratio; PARISK, Plaque At RISK; RR, relative risk; TIA, transient ischemic attack; TPA, total plaque area; US, ultrasonography.

Related dimensional measures were evaluated in the Tromsø Study [25,26], where total plaque area predicted myocardial infarction and ischemic stroke, and in the Northern Manhattan Study [24], where maximal plaque thickness was associated with combined vascular events. Nicolaides et al. [42] further showed that an assessment incorporating plaque area, thickness, and anatomical distribution was associated with long-term atherosclerotic cardiovascular outcomes.

Cross-sectional studies provided complementary evidence on plaque detection and concurrent disease burden: ICEBERG [29] highlighted the frequent presence of carotid plaque among individuals classified as being at low or intermediate cardiovascular risk, whereas the CPL Registry [28] showed an association between maximal plaque length and coronary artery disease severity. Regional studies such as ELSA-Brasil [36] and CARMELA [34,35] contributed data on plaque prevalence and cardiovascular risk factor associations in Latin American populations, although prospective outcome data remain limited.

MRI- and computed tomography angiography-based studies in symptomatic populations provided additional evidence on plaque composition and vulnerability but did not evaluate ultrasound-based CPS directly [40,43]. Finally, H3Africa AWI-Gen, which assessed carotid intima-media thickness but not plaque score, highlights the continuing lack of CPS-specific evidence from African populations [38].

Overall, these studies support the clinical relevance of carotid plaque burden, but their measures should not be interpreted as interchangeable CPS systems. The strongest prognostic evidence therefore derives from prospective cohorts with adjudicated cardiovascular outcomes, whereas cross-sectional and regional studies primarily inform plaque prevalence, methodological feasibility, and external validity. The absence of standardized scoring protocols, externally validated thresholds, and prospective evidence linking CPS-guided decisions to improved outcomes remains a major barrier to routine clinical implementation.

### 4.4 Plaque Burden and Vulnerability: Complementary but Distinct Dimensions

Although CPS quantifies the anatomical distribution and extent of carotid atherosclerosis, it does not capture plaque composition or features associated with biological vulnerability. High-risk characteristics, including intraplaque hemorrhage, a large lipid-rich necrotic core, a thin or ruptured fibrous cap, and plaque ulceration, have been associated with an increased risk of ischemic events, often beyond that explained by luminal stenosis alone [44,45,46]. In the Rotterdam Study MRI substudy, intraplaque hemorrhage was independently associated with incident stroke and coronary heart disease [22]. Similarly, the PARISK study showed that intraplaque hemorrhage and total plaque volume were associated with recurrent ipsilateral stroke or transient ischemic attack in patients with symptomatic mild-to-moderate carotid stenosis [43].

Plaque burden and vulnerability should therefore be regarded as complementary but non-interchangeable dimensions of atherosclerotic risk. CPS reflects the distribution and cumulative extent of disease and can be assessed using conventional ultrasonography, whereas detailed characterization of plaque composition generally requires advanced imaging, particularly MRI or CT [11]. However, most evidence on vulnerability features derives from patients with symptomatic carotid disease or established stenosis and cannot be directly extrapolated to asymptomatic primary prevention populations [46,47]. Moreover, no prospective study has demonstrated that adding vulnerability imaging to CPS improves risk prediction, treatment selection, or clinical outcomes. Accordingly, current evidence supports the conceptual complementarity of plaque burden and vulnerability but not their routine integration into a combined clinical scoring strategy.

## 5. Integration Into Guidelines, Consensus Statements, and Clinical Practice

Current cardiovascular prevention guidelines do not endorse CPS as a stand-alone screening or treatment-allocation strategy. Instead, carotid plaque imaging is generally positioned as a selective risk modifier, whereas CAC remains the preferred imaging method when conventional risk estimation leaves preventive treatment decisions uncertain (Table 3, Ref. [1,10,48,49,50,51,52,53,54,55]).

**Table 3.** **Guideline and consensus recommendations on carotid plaque imaging and coronary artery calcium for cardiovascular risk assessment**.

Guideline or document, year	Position on carotid plaque imaging and CPS	Position on CAC
ESC Guidelines on Cardiovascular Disease Prevention, 2016 [48]	Ultrasound detection of carotid plaque may be considered as a risk modifier in selected individuals (Class IIb, Level B). No CPS-specific threshold was recommended.	CAC may be considered as a risk modifier in selected individuals (Class IIb, Level B).
ACC/AHA Guideline on the Primary Prevention of Cardiovascular Disease, 2019 [49]	Routine carotid plaque imaging was not recommended for cardiovascular risk assessment, and no specific role was assigned to CPS.	CAC measurement may be considered in selected borderline- or intermediate-risk adults when treatment decisions remain uncertain (Class IIa, Level B-NR).
ASE Recommendations for the Assessment of Carotid Arterial Plaque by Ultrasound, 2020 [10]	Supports a stepwise approach integrating conventional risk assessment with carotid plaque imaging. Plaque burden assessment, including CPS, is considered more informative than isolated CIMT measurement.	Recognizes CAC as an established risk-reclassification tool and positions carotid plaque imaging as a complementary or alternative approach in selected individuals.
ESC Guidelines on Cardiovascular Disease Prevention, 2021 [50]	Carotid plaque assessment by ultrasound may be considered as a risk modifier when CAC scoring is unavailable or not feasible. No standardized CPS threshold was proposed.	CAC scoring may be considered to improve risk classification around treatment decision thresholds (Class IIb, Level B).
CCS Guidelines for the Management of Dyslipidemia, 2021 [51]	Carotid plaque imaging and CPS are not included among the principal recommended risk modifiers.	CAC greater than 0 may support treatment decisions in selected intermediate-risk individuals.
ESCR Consensus Statements on Carotid Artery Imaging, 2023 [52,53]	Supports standardized CT- and MRI-based assessment of plaque morphology, composition, and vulnerability, primarily in patients with established carotid disease and in specialized settings. Does not provide CPS-based treatment thresholds.	CAC is not a primary focus of the documents.
ESC Council on Stroke Clinical Consensus Statement, 2023 [54]	Supports integration of plaque burden and vulnerability imaging into multidisciplinary assessment of patients with carotid stenosis. The document does not endorse CPS for routine primary-prevention screening.	CAC is not a primary focus of the document.
AHA/ASA Guideline for the Primary Prevention of Stroke, 2024 [55]	Recommends against routine screening of the general population for asymptomatic carotid stenosis. Advanced plaque characterization is identified as an area requiring further research.	CAC is not a primary focus of the guideline.
ACC/AHA Guideline on the Management of Dyslipidemia, 2026 [1]	Recognizes carotid plaque as evidence of elevated ASCVD risk, including among individuals with CAC = 0, encourages initiation of LLT in patients with carotid plaque, although without formal Class of Recommendation or specific LDL-C targets. Routine carotid ultrasound screening and CPS-specific thresholds are not recommended.	Selective CAC assessment is recommended for risk reclassification when lipid-lowering decisions remain uncertain (Class 1, Level B-R).

Abbreviations: ACC, American College of Cardiology; AHA, American Heart Association; ASA, American Stroke Association; ASCVD, atherosclerotic cardiovascular disease; ASE, American Society of Echocardiography; B-NR, Level of Evidence B, nonrandomized; B-R, Level of Evidence B, randomized; CAC, coronary artery calcium; CCS, Canadian Cardiovascular Society; CIMT, carotid intima-media thickness; CPS, carotid plaque score; CT, computed tomography; ESC, European Society of Cardiology; ESCR, European Society of Cardiovascular Radiology; LDL-C, low-density lipoprotein cholesterol; LLT, lipid-lowering therapy; MRI, magnetic resonance imaging.

European recommendations have progressively refined the role of carotid imaging in cardiovascular risk assessment. The 2016 European Society of Cardiology (ESC) prevention guidelines stated that ultrasound assessment of carotid plaque may be considered as a risk modifier in selected individuals, while discouraging routine measurement of CIMT for risk prediction [48]. The 2021 ESC prevention guidelines subsequently emphasized CAC as the best-established imaging modality for risk reclassification and stated that carotid plaque assessment by ultrasound may be considered when CAC scoring is unavailable or not feasible [50]. Neither document endorsed routine population screening or proposed a specific CPS-based treatment threshold.

More recent European consensus documents have broadened the focus from plaque detection to plaque morphology and vulnerability. The ESC clinical consensus statement on carotid atherosclerotic disease incorporates stenosis severity, plaque burden, and vulnerability characteristics into individualized stroke-risk assessment [54]. Similarly, the 2023 European Society of Cardiovascular Radiology consensus documents support standardized CT- and MRI-based assessment of plaque morphology and composition [52,53]. However, these documents primarily address patients with established carotid disease, cerebrovascular symptoms, or specialist imaging indications and should not be interpreted as supporting routine CPS screening in asymptomatic primary prevention populations.

Among imaging societies, the 2020 American Society of Echocardiography recommendations provide the most detailed framework for ultrasound-based carotid plaque assessment [10]. The document favors assessment of plaque burden over isolated CIMT and recommends standardized evaluation of the distal common carotid artery, carotid bulb, and proximal internal carotid artery. Nevertheless, it also acknowledges the need for further standardization of plaque definitions, acquisition protocols, scoring methods, and clinically actionable thresholds.

North American prevention guidelines continue to give greater weight to CAC. The 2019 American College of Cardiology (ACC)/American Heart Association (AHA) primary prevention guideline recommended CAC assessment in selected borderline- or intermediate-risk adults when treatment decisions remained uncertain but did not provide specific recommendations regarding carotid plaque imaging or CPS [49]. The 2026 ACC/AHA dyslipidemia guideline further strengthens the role of selective CAC assessment in risk reclassification and treatment personalization [1]. Carotid plaque is recognized as evidence of subclinical atherosclerosis that may support initiation or intensification of lipid-lowering therapy in individualized decision-making, but routine carotid ultrasound screening and CPS-specific treatment thresholds are not recommended [1]. The 2021 Canadian Cardiovascular Society guideline similarly endorses CAC as a risk modifier in selected intermediate-risk individuals but does not include CPS among its principal recommended imaging tools [51].

The heterogeneous positions of professional societies reflect differences in the underlying evidence. CAC has shown the strongest and most consistent performance for predicting coronary outcomes and improving risk reclassification [17,19], whereas carotid plaque assessment may provide complementary information on systemic and cerebrovascular atherosclerotic burden [11,17]. Carotid imaging may be particularly informative when estimated risk appears discordant with the clinical profile, when plaque is detected incidentally, or when CAC scoring is unavailable, impractical, or not acceptable to the patient [10]. Studies showing risk misclassification by SCORE2 according to plaque status further support this potential role [5,12].

However, evidence that CPS improves statistical risk prediction should be distinguished from evidence that its use improves clinical outcomes. No randomized trial has demonstrated that initiating or intensifying preventive therapy according to CPS reduces cardiovascular events compared with management based on conventional risk assessment or CAC. Moreover, existing studies have used different scoring systems and thresholds. CPS values should therefore be interpreted only within the scoring method and population in which they were derived.

In current practice, CPS may be considered a complementary risk modifier in selected individuals with borderline or intermediate estimated risk when its result is expected to influence shared treatment decisions. It should not replace validated risk equations, be regarded as equivalent to CAC, or independently determine the initiation of antiplatelet therapy or a specific low-density lipoprotein cholesterol (LDL-C) target [1,49]. Routine screening of the general population cannot currently be recommended [55].

Overall, the guideline landscape supports a graded interpretation of imaging evidence: CAC has the strongest and most standardized role [1], carotid plaque assessment has a selective complementary role [10,50], and formal CPS-guided treatment remains investigational.

## 6. Limitations and Gaps in Evidence

The methodological heterogeneity of CPS definitions, scoring approaches, plaque definitions, ultrasound protocols, study populations, and geographic representation has been discussed in detail in Section 4. The present section therefore focuses on the interventional and implementation gaps that currently prevent CPS from being translated from a prognostic marker into a clinically validated tool for guiding preventive treatment decisions (Table 4, Ref. [10,12,13,15,17,19,22,27,30,31,32,33,34,35,36,37,38,39,40,43,44,45,46,47,50,56,57,58,59,60,61,62,63]).

**Table 4.** **Main evidence gaps and research priorities for the clinical application of carotid plaque score**.

Domain	Current evidence	Principal gap	Research priority
CPS-guided treatment allocation [12,17,27,56]	Prognostic associations supported by observational cohorts; modeled treatment effects available from MESA and ARIC	No randomized evidence that CPS-guided therapy improves cardiovascular outcomes	Randomized comparison of usual risk-based care versus CPS-guided preventive treatment
Aspirin allocation [56]	Observational modeling suggests a more favorable benefit-risk profile in selected individuals with greater plaque burden	Bleeding risk and net clinical benefit have not been prospectively evaluated using CPS	Trials restricted to carefully selected populations with prespecified ischemic and bleeding endpoints
Statin therapy [57,58,59,60]	Imaging studies suggest slower plaque progression and more stable plaque composition	No validated CPS threshold for initiating or intensifying lipid-lowering therapy	Evaluate CPS-guided statin intensity and LDL-C targets in borderline- and intermediate-risk individuals
PCSK9 inhibitors [61,62,63]	Small imaging studies suggest favorable changes in plaque composition and neovascularization	Limited sample sizes, surrogate outcomes, and no CPS-guided treatment studies	Larger trials linking plaque changes to clinical outcomes
Comparison with CAC [17,19,50]	CAC has stronger validation and guideline endorsement; carotid plaque may provide complementary information	No randomized comparison of CPS-guided and CAC-guided strategies	Pragmatic head-to-head trials including feasibility and cost-effectiveness
Scoring and acquisition protocols [10,12,13,15,30]	Multiple segment-based and dimensional approaches are available	No universally accepted CPS definition, protocol, or treatment threshold	International consensus on plaque definition, anatomical coverage, scoring, and reporting
Clinical implementation [10,37]	Carotid ultrasound is widely available and does not involve ionizing radiation	Limited integration with risk calculators, variable operator training, and uncertain reproducibility in routine care	Standardized training, automated reporting, implementation studies, and quality assurance
External validity [31,32,33,34,35,36,37,38,39]	Most prognostic evidence derives from European and North American cohorts	Limited prospective outcome data from Asian, African, and Latin American populations	External validation using standardized CPS protocols in geographically diverse populations
Plaque vulnerability [22,40,43,44,45,46,47]	MRI- and CT-defined high-risk features predict cerebrovascular events	Uncertain added value beyond CPS in asymptomatic primary prevention	Prospective evaluation of combined plaque burden and vulnerability strategies

Abbreviations: ARIC, Atherosclerosis Risk in Communities; CAC, coronary artery calcium; CPS, carotid plaque score; CT, computed tomography; LDL-C, low-density lipoprotein cholesterol; MESA, Multi-Ethnic Study of Atherosclerosis; MRI, magnetic resonance imaging; PCSK9, proprotein convertase subtilisin/kexin type 9.

### 6.1 Absence of CPS-Guided Interventional Evidence

To date, no randomized trial has tested whether initiating or intensifying preventive treatment according to CPS improves cardiovascular outcomes compared with management based on conventional risk assessment. The available evidence derives predominantly from observational cohorts and post hoc modeling analyses.

A harmonized analysis of MESA and ARIC suggested that carotid plaque burden may help identify individuals with a more favorable benefit-risk profile for aspirin therapy in primary prevention. Among participants with CPS ≥2 and an estimated 10-year atherosclerotic cardiovascular disease risk between 5% and 20%, the modeled number needed to treat was lower than the estimated number needed to harm [56]. However, this analysis was based on observational event rates and modeled treatment effects rather than prospective treatment allocation. It therefore provides hypothesis-generating evidence but cannot establish that CPS-guided aspirin use improves net clinical benefit.

The absence of randomized evidence is not unique to carotid imaging. Although CAC has substantially stronger validation and guideline endorsement, randomized studies of multimodal imaging-based cardiovascular screening have not consistently demonstrated reductions in mortality or major cardiovascular events. In DANCAVAS [64] and DANCAVAS II [65], comprehensive screening strategies incorporating computed tomography and CAC assessment did not significantly reduce their primary clinical outcomes, and DANCAVAS II reported an increased risk of severe bleeding. Because these interventions included several diagnostic and therapeutic components, their findings cannot be attributed specifically to CAC-guided management. Nevertheless, they illustrate the difficulty of demonstrating that improved detection of subclinical disease translates into better clinical outcomes and highlight the need to evaluate both preventive benefits and treatment-related harms in future CPS-guided trials.

### 6.2 Therapeutic Evidence From Plaque Imaging Studies

Imaging studies provide biological support for the use of carotid plaque assessment to evaluate treatment-associated changes in atherosclerotic burden and composition, but they do not establish the clinical utility of CPS-guided therapy. Statins have been associated with slower plaque progression, reductions in CIMT, increased plaque echogenicity, and a shift from lipid-rich toward more calcified plaque characteristics generally interpreted as greater stability [57,58,59]. In the Rotterdam MRI substudy, statin use was associated with a lower prevalence of lipid-rich plaque components and a higher prevalence of calcification [57,59,60]. Nevertheless, no randomized trial has evaluated whether statin initiation, treatment intensity, or LDL-C targets should be selected according to CPS.

Preliminary imaging studies have also examined proprotein convertase subtilisin/kexin type 9 (PCSK9) inhibitors. Small studies using MRI or contrast-enhanced ultrasound have reported favorable changes in lipid-rich necrotic core, intraplaque neovascularization, or total plaque area after treatment with evolocumab or other PCSK9 inhibitors [61,62,63]. However, these studies were generally small, used surrogate imaging outcomes, and were not designed to determine whether plaque changes translated into fewer cardiovascular events. CPS has not been used as a validated treatment-allocation criterion or as the primary outcome of an adequately powered clinical trial in this setting.

Trials such as ECST-2 [66], CREST-2 [67], and ACTRIS (NCT02841098) have evaluated or are evaluating whether clinical and imaging characteristics can identify patients with established carotid stenosis who benefit from revascularization. These studies may clarify the role of plaque imaging in carotid disease management, but they address a different clinical question from CPS-guided primary prevention in asymptomatic individuals without significant stenosis. Their findings should therefore not be extrapolated directly to the use of CPS for cardiovascular risk modification.

### 6.3 Priorities for Future CPS Trials

Future trials should determine whether CPS provides actionable information beyond conventional risk equations and CAC rather than merely demonstrating statistical associations. A clinically relevant design would enroll individuals at borderline or intermediate cardiovascular risk for whom the decision to initiate or intensify preventive therapy remains uncertain. Participants could be randomized to usual risk-based management or to a CPS-guided strategy in which a prespecified and externally validated scoring method and threshold trigger treatment intensification. Such trials should use a standardized ultrasound protocol, a reproducible CPS definition, blinded endpoint adjudication, and clinically relevant outcomes, including MACE, ischemic stroke, treatment-related bleeding, and adverse effects. The intervention should also specify in advance which treatment decisions are modified by CPS, such as statin initiation or intensification, rather than leaving treatment escalation to clinician discretion. Comparisons with CAC-based strategies would be informative, particularly where CAC is unavailable or not feasible, or when carotid ultrasound is already being performed for another clinical indication. Cost-effectiveness, operator reproducibility, feasibility in routine care, patient acceptability, and the consequences of incidental findings should be evaluated alongside cardiovascular outcomes. Prespecified subgroup analyses should assess whether the clinical value of CPS differs according to age, sex, ethnicity, baseline cardiovascular risk, and CAC status. Until these data are available, CPS should be regarded as a complementary risk modifier rather than an independently validated treatment-allocation tool.

## 7. Future Perspectives

The future clinical development of CPS will depend on addressing four interrelated priorities: standardization of acquisition and scoring, reduction of operator dependence, integration of plaque burden with vulnerability characteristics, and incorporation of CPS into validated clinical risk pathways. Progress in each of these areas will require prospective evaluation before broader clinical implementation.

### 7.1 Standardization of Scoring Protocols and Reporting

The most immediate priority is the development of a standardized CPS protocol. The American Society of Echocardiography recommends that plaque assessment include the distal 1 cm of the common carotid artery, the carotid bulb, and the proximal 1 cm of the internal carotid artery bilaterally [10]. However, this protocol has not been universally adopted, and substantial variability remains in the anatomical segments examined, plaque definitions, measurement methods, and reporting thresholds.

A formal consensus initiative should define a reference plaque definition, standardized anatomical coverage, the distinction between segment-count and dimension-based approaches, and minimum requirements for image acquisition, operator training, reproducibility assessment, and quality assurance. Standardized reporting should also specify the scoring method used rather than reporting a CPS value without methodological context. Such standardization is essential for multicenter validation, comparison across studies, and the development of clinically actionable thresholds.

The Plaque Reporting and Data System (Plaque-RADS) provides a structured framework for describing plaque morphology and composition across ultrasound, CT, and MRI [68,69]. However, Plaque-RADS and CPS evaluate different dimensions of carotid disease. CPS quantifies the extent and distribution of plaque, whereas Plaque-RADS focuses primarily on morphology and vulnerability. They should therefore not be considered interchangeable scoring systems. Whether their integration into a combined burden-and-vulnerability framework improves risk prediction or clinical decision-making requires prospective evaluation.

### 7.2 Artificial Intelligence-Assisted Plaque Assessment

Artificial intelligence may reduce operator dependence by automating plaque detection, segmentation, measurement, and classification. Recent meta-analyses have reported generally high diagnostic performance for image-based carotid plaque detection and vulnerability classification [70,71,72]. A multicenter diagnostic study has also reported promising performance for deep learning-based classification of carotid plaque vulnerability using contrast-enhanced ultrasound video [73].

Nevertheless, most available models have been developed using retrospective datasets, and external validation remains limited [70,71]. Differences in ultrasound equipment, acquisition settings, patient populations, plaque definitions, and model architectures may reduce transportability across centers. Moreover, current studies have mainly assessed diagnostic accuracy rather than prognostic performance, effects on clinical decisions, or patient outcomes [70,71].

A stepwise validation pathway is therefore needed. Initial algorithm development should be followed by internal validation, independent external validation across equipment manufacturers and clinical settings, prospective multicenter evaluation, and implementation studies within routine workflows. Ultimately, artificial intelligence-assisted CPS should be compared with conventional operator-based scoring in terms of reproducibility, prognostic discrimination, feasibility, workflow efficiency, cost-effectiveness, and clinical outcomes.

### 7.3 Integration of Plaque Burden and Vulnerability

Current CPS systems quantify plaque distribution or dimensions but do not systematically incorporate plaque composition or vulnerability characteristics. Advanced imaging modalities can identify high-risk features such as intraplaque hemorrhage, lipid-rich necrotic core, fibrous cap disruption, ulceration, and neovascularization [58].

MRI allows detailed non-invasive characterization of carotid plaque composition [58,74], whereas CT provides high spatial resolution for the assessment of calcification, ulceration, and plaque morphology [74,75]. Contrast-enhanced ultrasound may offer a more accessible method for evaluating intraplaque neovascularization using the same imaging platform employed for conventional CPS assessment [76,77]. However, these techniques require additional examination time, technical expertise, and costs. CT also involves ionizing radiation and may require contrast administration, while the acquisition and interpretation of vulnerability features remain incompletely standardized across modalities.

One potential research strategy would combine a simple ultrasound-based CPS for initial assessment of plaque burden with selective advanced imaging in individuals with high plaque burden, discordant clinical risk, or suspected high-risk morphology. This sequential approach may be more feasible than performing advanced plaque characterization in all individuals. However, the incremental prognostic value, clinical utility, and cost-effectiveness of combining CPS with vulnerability imaging remain unproven [78].

### 7.4 Integration With Risk Models and Clinical Pathways

CPS would most plausibly provide clinical value as part of an established risk-assessment pathway rather than as an isolated screening test [10]. Potential applications include individuals with borderline or intermediate estimated risk, patients in whom conventional risk estimates appear discordant with the clinical profile, and individuals undergoing carotid ultrasound for another indication in whom plaque is detected incidentally [5,79].

Future studies should evaluate whether adding CPS to SCORE2, PREVENT, or the Pooled Cohort Equations improves not only statistical measures of discrimination and reclassification but also therapeutic decision-making, patient adherence, cost-effectiveness, and clinical outcomes [5,12,14]. Comparisons with CAC-based pathways are particularly important to determine whether CPS provides complementary information or primarily represents an alternative when CT is unavailable, unsuitable, or not acceptable to the patient [11,17,80].

A practical implementation pathway could include standardized acquisition, automated or assisted plaque scoring, electronic integration with established risk calculators, and structured reports that clearly identify the scoring method and plaque distribution. Any proposed risk categories or treatment thresholds should first be prospectively derived and externally validated. This approach should subsequently be evaluated in multicenter clinical and implementation studies before broader adoption. Until these steps are completed, CPS should remain a complementary imaging-based risk modifier rather than a stand-alone screening or treatment-allocation tool.

## 8. Conclusions

The carotid plaque score is a rapid, non-invasive, and relatively inexpensive measure of subclinical atherosclerotic burden that may provide incremental prognostic information beyond conventional risk models, particularly for ischemic stroke and MACE. However, the available evidence remains predominantly observational, and the clinical interpretation of CPS is limited by heterogeneous plaque definitions, scoring methods, anatomical protocols, and proposed thresholds. Further progress requires standardized acquisition and reporting protocols, external validation across diverse populations, and prospective studies evaluating whether CPS-guided strategies influence treatment decisions and improve clinical outcomes. Until such evidence is available, CPS should be considered a complementary risk modifier in selected individuals, rather than a stand-alone screening or treatment-allocation tool. The principal challenge is therefore to translate its prognostic value into a standardized, reproducible, and clinically actionable role within preventive care.
